# Effective Treatment of Rosacea and Other Vascular Lesions Using Intense Pulsed Light System Emitting Vascular Chromophore-Specific Wavelengths: A Clinical and Dermoscopical Analysis

**DOI:** 10.3390/jcm13061646

**Published:** 2024-03-13

**Authors:** Domenico Piccolo, Irene Fusco, Tiziano Zingoni, Claudio Conforti

**Affiliations:** 1 Novea Skin Center—Dermo Aesthetic Laser Centers, 67051 Avezzano, Italy; domenico.piccolo.skincenters@gmail.com; 2 El.En. Group, 50041 Calenzano, Italy; t.zingoni@elen.it; 3 IDI-IRCCS, Dermatological Research Hospital, 00167 Rome, Italy; claudioconforti@yahoo.com

**Keywords:** IPL, dermatoscopy, vascular lesions, rosacea

## Abstract

**Background**: Facial telangiectasias is a prevalent cosmetic disorder that can be associated with several conditions such as rosacea. IPL (intensity pulsed light) therapy is commonly used for the treatment of vascular lesions. This study tested the efficacy of an IPL system emitting selected vascular chromophore-specific wavelengths in the range of 500–1200 nm for the treatment of vascular lesions. **Materials and Methods**: A total of 39 patients affected by different vascular lesions on their face were enrolled. The procedure consisted of three treatment sessions, spaced 1 month apart, using the IPL system with a 500–677 and 854–1200 nm filter. Follow-up was performed at 21–90 days (3 weeks–3 months) after the last IPL session. Three-dimensional and dermoscopic clinical photographs were captured and evaluated using a five-point scale. Adverse events were checked. **Results**: In total, 21 patients achieved excellent improvement, 13 patients achieved good improvement, 3 patients achieved moderate improvement, 2 patients achieved mild improvement, and 0 patients achieved no improvement, with an overall good response to treatment. The photographic evaluation showed good results as soon as 3 days after the last IPL session. Relevant side effects were absent. **Conclusions**: The study device may represent a successful treatment to improve vascular lesions that are resistant to laser therapy.

## 1. Introduction

Rosacea is an inflammatory skin condition that mostly impacts the face’s central areas, and it represents a prevalent chronic dermatosis that affects around 5.46% of people worldwide [1]. The percentage of affected areas varies from 1% to 22%. 

This condition typically strikes both males and females equally, usually starting in middle to late adulthood. Northern European populations are primarily affected by the disease [2]. 

In the initial phases of rosacea, frequent flushing or blushing as well as sporadic episodes of facial redness may be present. A family history of rosacea, a tendency to blush, or both may increase an individual’s risk of obtaining the condition. In these cases, lifestyle alterations are advised to reduce triggers. Although individual patients may have different triggers, the most common ones are alcohol, spicy meals, sun exposure, and stress [3,4]. 

Rosacea is persistent and subject to relapses. Its prevalent dermal manifestations include pustules, papules, chronic centrofacial erythema, recurrent flushing, phymatous alterations, a range of ocular symptoms, and telangiectasias.

Among these, facial telangiectasias is a common cosmetic issue, with mild or limited lesions seen in 92% of adult Caucasians [5] that may be associated with rosacea condition, with Civatte poikiloderma, or to a number of variables such as genetic susceptibility, gravity, pregnancy, and trauma [6,7]. People with lighter skin phototypes and an age range between 30 and 50 years have a higher probability of being affected [8]. 

Numerous treatments, including topical lotions, systemic therapy lasers, and light devices, have been suggested to address this cosmetic issue, with a range of results [9,10]. 

Patients with rosacea can have a wide range of skin characteristics, varying in severity. Treatment options range from moderate monotherapy to combination medicines that target one or more parts through additional mechanisms depending on the unique composition of clinical characteristics. Targeting specific phenotypes is frequently the goal of effective treatment [11].

Nowadays, alpha-adrenergic agonist-based topical gels, like brimonidine, are being considered as treatments for minor facial telangiectasias and erythrosis. Nevertheless, they have a transient impact that lasts for a maximum of 12 h and do not affect telangiectasias of a higher caliber [12,13].

Among systemic therapies, isotretinoin and oral beta-blockers are usually administered. However, isotretinoin may cause cheilitis, myalgia, dry mouth and lips, birth defects, epistaxis, and increased alanine aminotransferase and triglyceride levels. On the other hand, oral beta-blockers can cause hypotension, bradycardia, or dizziness [14].

Rosacea features, particularly telangiectasias and phymatous transformation, have been successfully managed with light therapy and lasers [15]. In order to target larger vessels and produce longer-lasting cosmetic results, laser and light sources have been suggested.

Long-pulsed neodymium-doped yttrium aluminum garnet laser (Nd: YAG), IPL (intensity pulsed light), potassium titanyl phosphate (KTP) laser, and pulsed dye laser (PDL) are the most commonly used laser treatments [15].

Indeed, several lasers, most notably the flashlamp-pulsed dye laser, have been clinically shown to be beneficial for treating facial vascular lesions. Nonetheless, individuals have expressed concern about post-treatment adverse effects such as severe purpura and changes in pigmentation [16].

Carbon dioxide (CO_2_) and erbium-doped yttrium aluminum garnet (Er:YAG) lasers are other useful treatments for phymatous rosacea. Features of telangiectasia such as flushing and erythema are responsive to light therapy treatment. Considering that the telangiectasia component is frequently resistant to other treatments, both PDL and IPL devices are effective and satisfy patients’ needs [11].

Fitzpatrick, Goldman, and Eckhouse illustrated how IPL, which emits multiple wavelengths simultaneously using an optical intercept filter to select the spectral output, can be used therapeutically [17]. Since the FDA approved IPL in 1995, many devices that allow for the modulation of energy fluence, spectral output, area size, and pulse modulation duration have been developed.

IPL has been recommended in the management of many conditions, such as rosacea and photoaging [18,19,20,21,22].

Several studies have demonstrated that IPL therapy is a commonly used treatment for telangiectasia and erythematous lesions [23,24]. With promising findings, IPL has been proposed to also treat erythrosis [25,26] and hemangioma, as reported in the study of Vladimir et al. [27], in which the IPL method outperformed conventional methods, particularly for flat hemangiomas. One major benefit of IPL treatment over standard therapies is the patient’s rapid recovery time. Furthermore, the appropriate choice of patient and the precise measurement of wavelength are crucial in every instance.

IPL generates an effective, broad-spectrum light pulse that is typically in the 400–1200 nm range, though different filters can be used to narrow the wavelength range and focus the light more closely on a particular target. Indeed, several studies have demonstrated the efficacy and advantages of selecting specific ranges of wavelengths though the use of various filters for the management of vascular lesions, as reported in the published work of Peterson et al. [28] in which an IPL narrowband “KTP/PDL-like” filter (525 nm and 585 nm) was successfully used to treat facial telangiectasias and non-facial telangiectasias with minimal downtime. 

On these bases, this study tested the effectiveness of an alternative form of therapy involving an intense pulsed light (IPL) system emitting selected vascular chromophore-specific wavelengths in the range of 500–1200 nm to treat vascular lesions such as telangiectasias, erythrosis, poikiloderma of Civatte, and late-stage rosacea, giving patients a more manageable outcome after treatment.

## 2. Materials and Methods

Patients affected by different vascular lesions (telangiectasia, rosacea, erythrosis, and poikiloderma) were included in this research. Excluded from the study were patients with a history of skin cancer, a history of hypersensitivity to light in the near-infrared wavelength region, patients taking immunosuppressants or anticoagulants, patients taking medications known to increase sensitivity to sunlight, such as sulfonamides, sulfonylureas, phenothiazines, and contraceptives, and patients who reported having been exposed to the sun in the three weeks prior to treatment. All patients provided an informed consent form regarding the procedure’s risks. 

Depending on the type of vascular lesions treated, the procedure consisted of 3 treatment sessions, spaced 1 month apart, covering an area of 15 mm × 13 mm, using the IPL system with a 500–677 and 854–1200 nm filter (Viridis handpiece of Luxea, DEKA M.E.L.A., Calenzano, Italy). The following parameters were set: 1–2 passes with Fluence ranging between 13 and 39 J/cm^2^ at 15–50 ms pulse duration and a frequency ranging between 0.2 and 1 Hz. Throughout every procedure, internal skin flow cryogen cooling at 5 °C was maintained. No subcutaneous injection or anesthetic cream were used because all patients felt that the pain was tolerable. A moderate redness and closure of the telangiectasias indicated the treatment’s end point. Up until clinical follow-up, patients were required to apply an SPF 50 protection cream every morning; follow-up was carried out at 21–90 days (3 weeks–3 months) after the last IPL session. 

Three-dimensional (3D) clinical photographs of the face were captured at three angles (left, center, and right) before treatments, immediately after, and 3 days, 21 days, 45 days, and 3 months after the last treatment session, using the 3D digital camera (Vectra H2, Canfield, OH, USA) for a more accurate and impartial assessment of the patient’s tissue texture and vascularity. The morphology, depth, and grade of the vascular lesions and their corresponding changes correlated with clinical outcome, and they were—more precisely—tracked for the ideal outcome, and a post-treatment evaluation of the laser treatments, using dermatoscopy (or epiluminescence microscopy (ELM)) (FotoFinder Systems GmbH 1000, Bad Birnbach, Germany), was performed at the same time points.

Given the lack of a defined scale for assessing facial telangiectasias, independent researchers assessed the images and assigned a 5-point scale rating (with 0 indicating no improvement/worsening of the condition; 1 indicating mild improvement of the condition; 2 indicating moderate improvement; 3 indicating good improvement; and 4 indicating excellent improvement/disappearance of the condition) [29].

Safety was monitored throughout the study by checking for any possible adverse effects such as blistering, scarring, hypopigmentation, or hyperpigmentation.

## 3. Results

A total of 39 patients (15 males and 24 females), ranging in age from 18 to 75 years old, with Fitzpatrick skin type II–III (48% type II and 52% type III), affected by different vascular lesions on their face area, were enrolled in this study. A total of 11 patients suffered from telangiectasia, 17 patients suffered from rosacea, 9 patients suffered from erythrosis, and 2 patients suffered from poikiloderma. 

**Figure 1:**
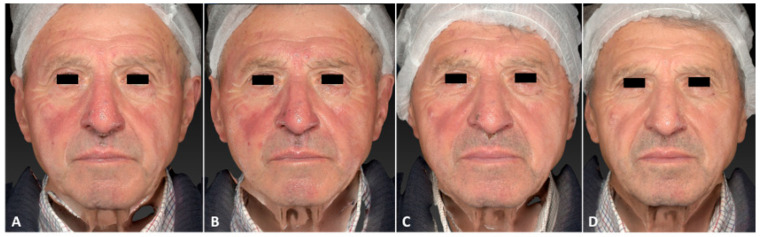
A 75-year-old male patient affected by rosacea with a Fitzpatrick skin type III before (**A**), immediately after (**B**), and following one IPL treatment session (**C**), and 3 months follow-up after the last IPL treatment session (**D**). The following parameters were used: single pass, Fluence 15 J/cm^2^, frequency 1 Hz, and pulse duration 20 ms.

**Figure 2:**
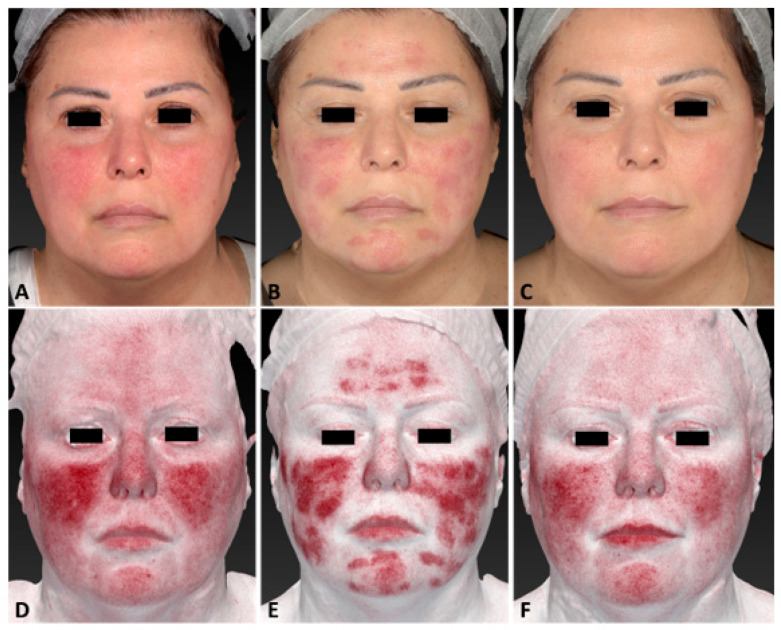
A 53-year-old female patient affected by rosacea with Fitzpatrick skin type II, before (**A**,**D**), immediately after (**B**,**E**), and 3 months follow-up after the last IPL treatment session (**C**,**F**). The following parameters were used: single pass, Fluence 30 J/cm^2^, frequency 0.6 Hz, and pulse duration 25 ms.

**Figure 3:**
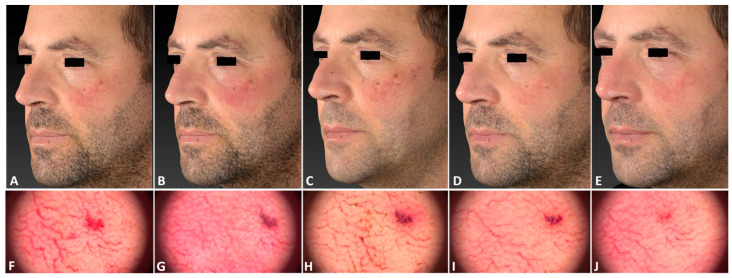
A 53-year-old male patient with telangiectasia localized on his left cheek, with a Fitzpatrick skin type III, before (**A**), immediately after (**B**), and 3 days (**C**), 11 days (**D**), and 21 days after (**E**) the IPL treatment session. The corresponding dermatoscopic analysis was carried out at the same follow-up times (**F**–**J**). The following parameters were used: single pass, Fluence 30 J/cm^2^, frequency 0.6 Hz, and pulse duration 25 ms.

**Figure 4:**
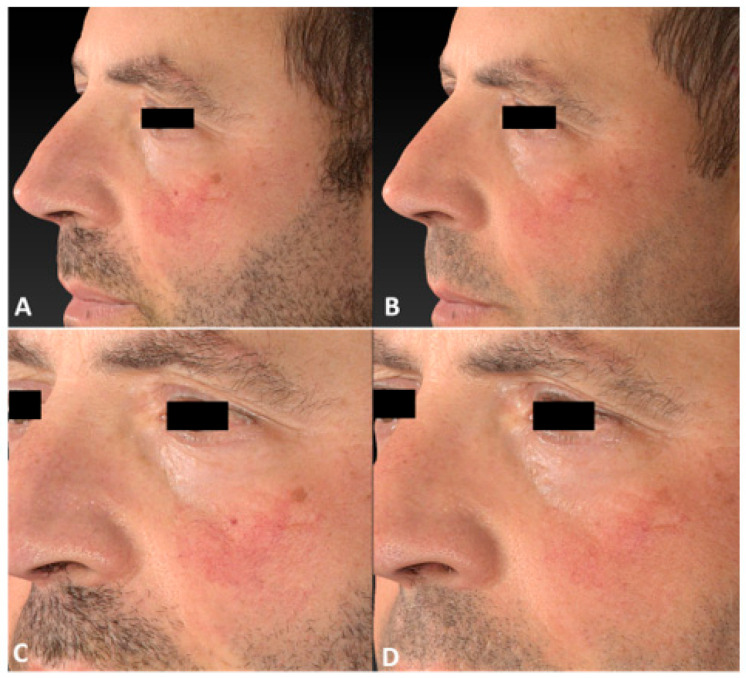
Left later view of the same patient’s cheek before (**A**,**C**), at 3 months follow-up after the last IPL treatment session, (**B**) and following 2 IPL treatments (**D**).

**Figure 5:**
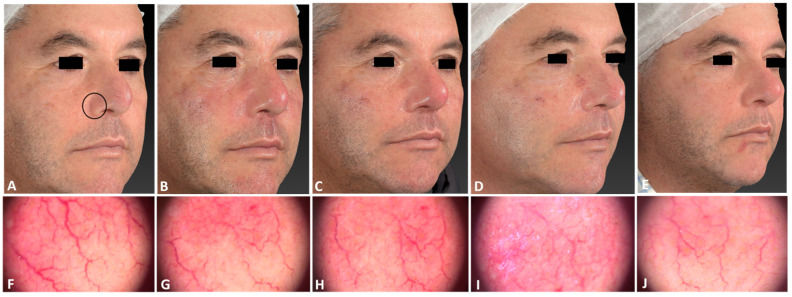
A male patient with telangiectasia localized on the right ala of his nose before (**A**), immediately after (**B**), 2 h after (**C**), 6 days after (**D**), and 21 days after (**E**) the IPL treatment session. The corresponding dermatoscopic analysis considering a precise skin area (black circle) was carried out at the same follow-up times (**F**–**J**). The following parameters were used: one single pass, Fluence 25 J/cm^2^, frequency 0.7 Hz, and pulse duration 30 ms.

**Figure 6:**
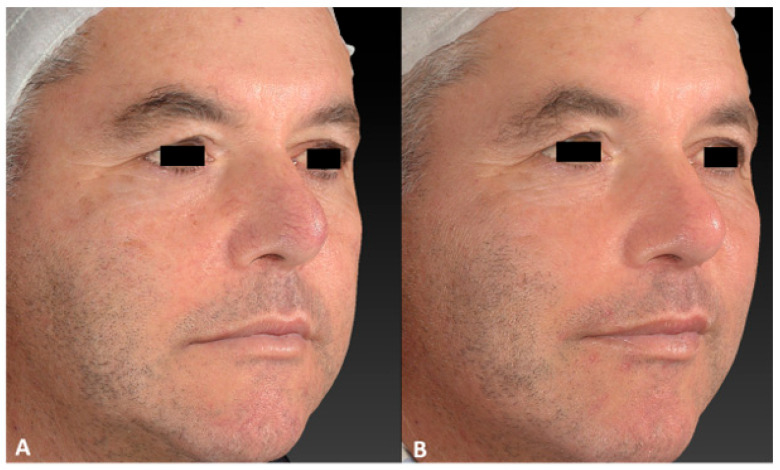
Digital images of the same male patient with telangiectasia localized on the right ala of his nose before (**A**) and after 3 IPL treatments (**B**). A marked reduction in and clearance of veins were observed.

**Figure 7:**
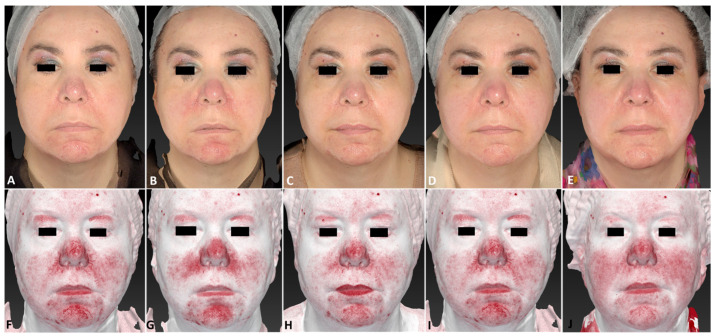
A 54-year-old female patient affected by telangiectasia localized on her nose area, including the right/left ala of her nose and chin before (**A**,**F**), immediately after (**B**,**G**), 3 days after (**C**,**H**), 21 days after (**D**,**I**), and 45 days after (**E**,**J**) the last IPL treatment session. The following parameters were used: left and right nose wings (double pass); back of nose (double pass); chin (single pass); Fluence 35 J/cm^2^; frequency 0.5 Hz; and pulse duration 40 ms.

**Figure 8:**
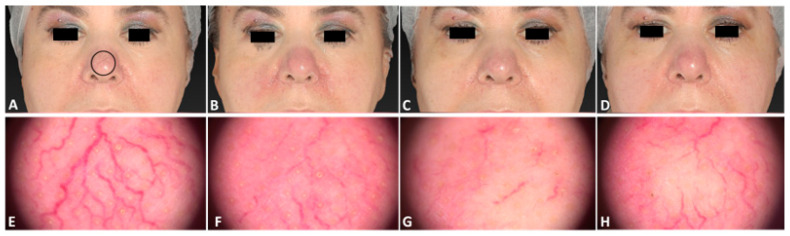
The same female patient affected by telangiectasia localized on her nose area before (**A**), immediately after (**B**), 3 days after (**C**), and 21 days after (**D**) the last IPL treatment session. The corresponding dermatoscopic analysis considering a precise skin area (black circle) was carried out at the same follow-up times (**E**–**H**).

**Figure 9:**
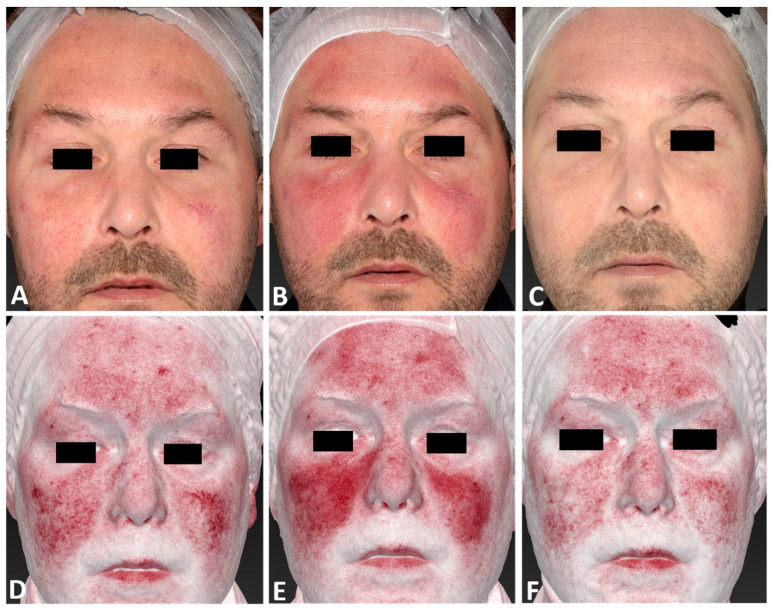
A 43-year-old male patient affected by telangiectasia on his cheeks and forehead, with Fitzpatrick skin type II before (**A**,**D**), immediately after (**B**,**E**), and 21 days after the last IPL treatment session (**C**,**F**). The following parameters were used: double pass, Fluence 35 J/cm^2^, frequency 0.5 Hz, and pulse duration 50 ms.

**Figure 10:**
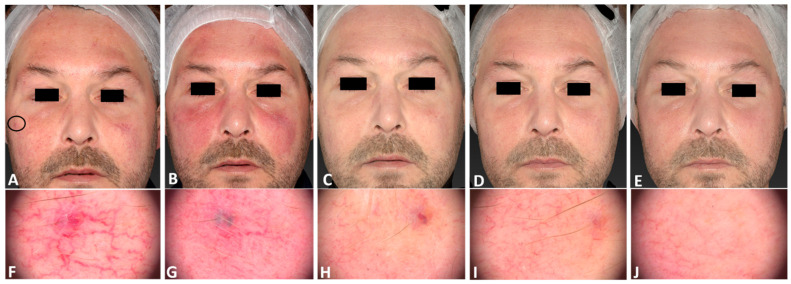
Male patient affected by telangiectasia on his cheeks and forehead before (**A**), immediately after (**B**), 15 days after (**C**), 21 days after (**D**), and after 2 IPL treatment sessions (**E**). The corresponding dermatoscopic analysis considering a precise skin area (black circle) was carried out at the same follow-up times (**F**–**J**).

**Figure 11:**
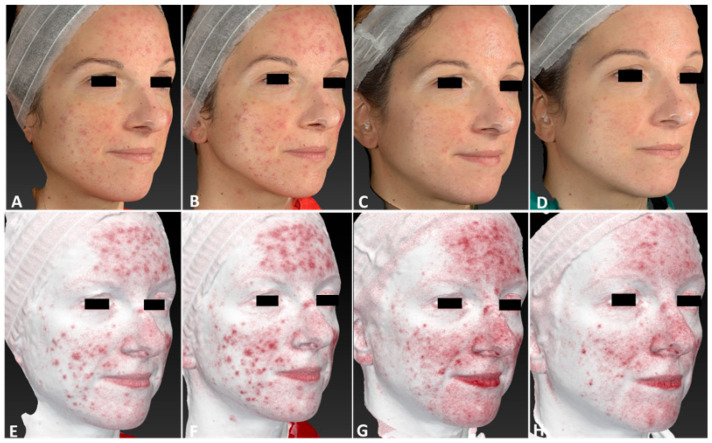
Left lateral view of a 35-year-old female patient affected by diffuse rosacea localized on her face, with Fitzpatrick skin type III, before (**A**,**E**), immediately after (**B**,**F**), and following one IPL treatment session (**C**,**G**), and at 3 months follow-up after the last IPL treatment session (**D**,**H**). The following parameters were used: double pass, Fluence 20 J/cm^2^, frequency 0.9 Hz, and pulse duration 30 ms.

**Figure 12:**
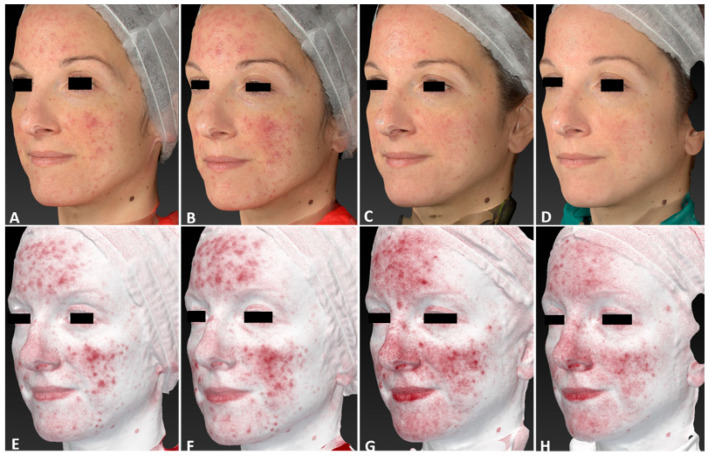
Right lateral view of a 35-year-old female patient affected by diffuse rosacea localized on her face, with Fitzpatrick skin type III, before (**A**,**E**), immediately after (**B**,**F**), and following one IPL treatment session (**C**,**G**), and at 3 months follow-up after the last IPL treatment session (**D**,**H**). The following parameters were used: double pass, Fluence 20 J/cm^2^, frequency 0.9 Hz, and pulse duration 30 ms.

**Figure 13:**
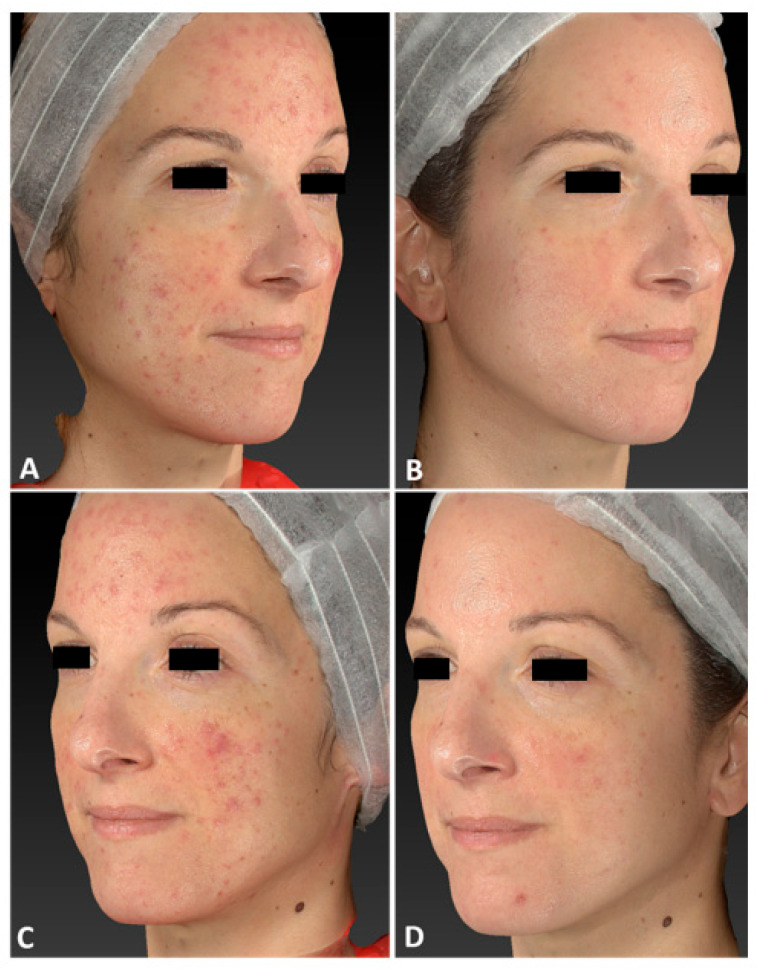
Right and left lateral view of the same female patient affected by diffuse rosacea localized on her face before (**A**,**C**) and at 3 months follow-up after the last IPL treatment session (**B**,**D**). The strongest reduction in lesion count and erythema was clinically observed.

The dermatologist evaluation comparing the pictures showed that 21 patients achieved excellent improvement, 13 patients achieved good improvement, 3 patients achieved moderate improvement, 2 patients achieved mild improvement, and 0 patients achieved no improvement, with a generally favorable response to therapy (Figure 1, Figure 2, Figure 3, Figure 4, Figure 5, Figure 6, Figure 7, Figure 8, Figure 9, Figure 10, Figure 11, Figure 12 and Figure 13). Furthermore, vascular treatment results acquired with the filter of the 3D digital Vectra system showed improvement in the patients’ original conditions, as clearly represented in Figure 2D–F.

When pigmented lesions (solar lentigines) were present, a paradoxical darkening was observed, with disappearance of the pigmented lesion within 21 days (as shown in Figure 3).

Both 3D clinical photographs and dermoscopic evaluation show good results in terms of vascular lesion clearance as soon as 3 days after the last IPL treatment session.

The strongest reduction in lesion count and erythema was clinically observed after three IPL treatment sessions (3 months follow-up after the last IPL treatment session). 

During the procedure, almost all patients reported no pain or minimal pain. Anesthesia was not required. All conditions lacked relevant side effects, such as crusts, blisters, atrophy, and scars. No post-inflammatory hyper/hypopigmentation was observed. Only a slight erythema was observed immediately after treatment, which spontaneously resolved.

## 4. Discussion

Patients with facial telangiectasias experience psychological problems in addition to cosmetic issues. Numerous laser therapies have been suggested; however, the medical literature now only includes a small number of combination treatments. IPL devices, as well as in combination with other therapies, have recently become extremely popular in managing these skin conditions [30]. According to actual investigations, intense pulsed light is extremely beneficial for treating vascular lesions like telangiectasias of the legs and face, spider nevi, rosacea, erythrosis, and poikiloderma of Civatte [31]. However, due to its high selectivity, current models of pulsed dye lasers continue to be the gold standard for treating port–wine stains, cherry angiomas, and other vascular anomalies. 

The results of a novel combination of IPL, 532 nm, and 1064 nm Nd YAG lasers for the treatment of facial erythrosis were recently analyzed by Bennardo et al. [32]. Their analysis showed that the sequential use of a 532/1064 nm Nd:YAG laser and IPL has proven to be particularly effective in the management of facial erythrosis and telangiectasias. Both processes are undoubtedly operator-dependent, so in order to reduce the incidence of side effects and boost efficacy, the therapy should be administered by a qualified researcher experienced with these instruments.

IPL devices, as opposed to lasers, generate a wide spectrum of wavelengths of noncoherent, polychromatic light. To target particular chromophores, they can alter their fluence, pulse duration, spot size, and filter type. Consequently, a wide variety of skin types and lesions can be treated with IPL equipment. Lesion clearance often depends on treatment frequency because the action of pulsed light is cumulative, usually requiring three to six treatments every 2 to 4 weeks to achieve the full clinical benefit [33]. 

Four fundamental concepts support the mechanism of IPL therapy: spot size, fluence, wave length, and pulse duration. Indeed, within the treatment area, competing chromophores should be considered by the healthcare professional when choosing a wavelength. For instance, the targeted chromophores found in a deep layer of the skin may be protected by melanin in the epidermis. This idea is especially crucial when using IPL on patients with darker skin tones because extra care needs to be taken to treat them properly and avoid dyschromias.

Greater pigmentation in the lesions may necessitate more treatment sessions. Because they are more difficult to penetrate, lesions located deeper within the dermis might also need a higher quantity of treatments. The IPL system’s light source emits wavelengths between 420 and 1400 nm and uses filters to target specific chromophores and improve penetration by limiting energy absorption by other chromophores. 

The idea of selective photothermolysis, which makes use of the hemoglobin absorption peak throughout its wavelength range, is the foundation of IPL treatment, also known as pulsed dye laser treatment. 

Since erythrocytes contain this pigment, blood arteries have a high concentration of it. 

Until it reaches the desired chromophore, light permeates the skin. The target structures can be intentionally heated and coagulated by selecting wavelengths that correspond to their respective maximum absorption levels. Photons are absorbed by endogenous or exogenous chromophores in the skin, which release thermal energy and heat the target tissue until it is destroyed by thermocoagulation [34,35,36].

IPL is associated with a shorter recovery period and lower equipment costs when compared to laser devices [37].

The benefits of using an IPL system include the ability to target many chromophores, lower costs, less complicated parameters that are more adaptable, and fewer adverse events [20]. The flexible broadband wavelength spectrum, which spans from 515 nm to 1200 nm, makes it possible to treat vessels at various depths.

Indeed, several filters were suggested in the early years of the new millennium to increase IPL’s efficacy for facial telangiectasias [38].

There were no differences in the results between the 595 nm laser and IPL with vascular filters when treating facial telangiectasias, according to recent research [38] that compared PDL and IPL with different filters (530–650 and 900–1200 nm).

Comparable outcomes have been noted by other researchers regarding telangiectasias of the face and body [39,40].

Campolmi et al. [41] validated the IPL source’s effectiveness by utilizing five distinct cut-off filters (500, 520, 550, 600, and 650 nm), permitting the emission of light in the wavelength range of 500 to 650 and 1200 nm. The greatest outcomes for vascular treatment are obtained with phototypes I–II, 500–520 nm wavelength filters, pulse durations up to 8 ms, selecting double pulses, adjusting the pulse delay based on the circumstances, and delivering greater fluences. 

A further investigation suggested treating face telangiectasias with a narrow band IPL light (500–600 nm) with positive outcomes [42].

Furthermore, additional research reports using vascular filters to increase IPL’s effectiveness are needed, such as the study of Peterson et al. [28], already cited in the Introduction section, or that reported by Luo et al. [43], in which a single IPL wavelength of 540 nm was also proposed as a treatment for late-stage rosacea and telangiectasias, with encouraging outcomes.

However, unlike PDL, IPL treatment for vascular lesions does not produce significant pain, which is one of the main discomforts experienced by the patient during PDL treatment, and it does not necessitate the use of anesthesia. In fact, local anesthesia should be avoided due to the probable constriction of the vessels, resulting in fewer targets [21]. Indeed, in our study, there was no subcutaneous injection or use of anesthetic lotion.

IPL has numerous advantages over alternative therapies that can help with rosacea or telangiectasias, including pulsed dye laser. Firstly, there are fewer side effects, either local or systemic (i.e., no postoperative purpura), due to the pulse delays that allow the skin to cool. The “purpura effect” is brought on by an unexpected rise in blood temperature that damages small dermal vessels, particularly with the older dye laser. The second benefit involves a bigger spot size, which is perfect for treating facial areas that require greater attention while reducing shortening treatment times and patient pain. Additionally, it avoids the honeycomb effect produced by the pulsed dye laser’s lower spot sizes [40].

The objective of this study was to supply sufficient power to raise blood vessel temperature to the coagulation threshold while preserving surrounding normal tissue. 

The design of the study device protocol includes variable pulse durations and the ability to perform multiple passes with a controlled delivery time so that the epidermis can cool in between pulses, ensuring that no epidermal damage occurs even with levels of fluency.

Additionally, in our study, the band between 677 and 854 nm is removed from the study laser system as it does not interfere with hemoglobin and it may instead produce additional heat, which could cause pain and other adverse effects.

Indeed, the flexibility to customize pulse duration makes IPL a powerful technique for treating vascular diseases. The choice of different filter settings provides for a broader selection of the vascular system’s color range [44].

Better visualization of blood flow and vessel dimensions could potentially enhance the outcomes of IPL treatment. Indeed, in the present investigation, participants underwent also to dermoscopy analysis both before and after IPL irradiation. The prediction inference by dermoscopy for selecting instances that will respond better has been discussed in a variety of scientific research [44]. More precise assessment of the morphology and patterns of vascularity, as well as monitoring for the ideal end point and post-therapy to allow for a predictable reaction and assessment of the laser treatments, are required. The diameter of the vessels also affects the laser’s result; smaller vessels respond less strongly than larger, frequently deeper vessels [45]. The color of lesions correlates with the size of the vessels, as shown by dermoscopy; pink lesions have smaller, deeper vessels, purple lesions have larger, deeper vessels, and red lesions have superficial vessels [46].

Because dermoscopic changes can be observed immediately after radiation exposure and the minimal effective fluence can be predicted, dermoscopy represents an essential clinical tool. For this reason, the dermoscopic observations examined immediately following irradiation are an essential clinical tool for laser surgeons as they enable them to estimate the least effective fluence and prevent skin damage.

Our findings, supported by the photographic investigation, showed good results in terms of reductions in all types of vascular lesions treated. Compared to laser treatment, the frequency and severity of cosmetic adverse effects are significantly lower. Furthermore, the presence of an external air-flow cryogen cooling mechanism in this research seems to be essential for minimizing scarring, reducing patient discomfort, and the overall enhancement of the procedure.

Our results are in agreement with other already published studies which showed the effectiveness of IPL for telangiectasia with a more tolerable post-treatment outcome [42,47,48,49], in which a significant reduction in the blood flow in this vascular condition was observed.

The absence of long-term side effects and bearable pain during the treatment makes this type of therapy a valuable solution for the resolution of a variety of vascular lesions. 

### Study Limitations

One limitation is the use of a multispectral camera operating in the NIR or MIR wavelengths, which can penetrate the skin to certain depths, providing information about the characteristics of the skin and other tissues in order to obtain information about blood flow, tissue oxygenation, and other skin properties. As a future goal for further studies, we will provide a more accurate imaging acquisition.

## 5. Conclusions

In conclusion, IPL is a safe method that minimizes costs, time, and adverse effects when treating vascular lesions such as rosacea. IPL therapy has made advances in treating these types of vascular lesions, benefiting both patients and doctors. For lesions that do not respond well to laser therapy, the study device might provide better outcomes. Developments of new and more precise parameters will aim to provide safer results, with optimal lesion resolution and improved patient satisfaction.

## Data Availability

Data that support the study findings are available on request from the corresponding author (I.F).

## References

[1] Rainer B.M., Kang S., Chien A.L. (2017). Rosacea: Epidemiology, pathogenesis, and treatment. Derm. Endocrinol..

[2] Gether L., Overgaard L., Egeberg A., Thyssen J. (2018). Incidence and prevalence of rosacea: A systematic review and meta-analysis. Br. J. Dermatol..

[3] Thiboutot D., Anderson R., Cook-Bolden F., Draelos Z., Gallo R.L., Granstein R.D., Kang S., Macsai M., Gold L.S., Tan J. (2020). Standard management options for rosacea: The 2019 update by the National Rosacea Society Expert Committee. J. Am. Acad. Dermatol..

[4] Parodi A., Paolino S., Greco A., Drago F., Mansi C., Rebora A., Parodi A., Savarino V. (2008). Small intestinal bacterial overgrowth in rosacea: Clinical effectiveness of its eradication. Clin. Gastroenterol. Hepatol..

[5] Meki´c S., Hamer M., Wigmann C., Gunn D., Kayser M., Jacobs L., Schikowski T., Nijsten T., Pardo L. (2020). Epidemiology and determinants of facial telangiectasia: A cross-sectional study. J. Eur. Acad. Dermatol. Venereol..

[6] Two A.M., Wu W., Gallo R.L., Hata T.R., Rosacea J. (2015). Rosacea: Part I. Introduction, categorization, histology, pathogenesis, and risk factors. Am. Acad. Dermatol..

[7] Katoulis A.C., Stavrianeas N.G., Georgala S., Bozi E., Kalogeromitros D., Koumantaki E., Katsambas A.D. (2005). Poikiloderma of Civatte: A clinical and epidemiological study. J. Eur. Acad. Dermatol. Venereol..

[8] Vissing A.-C.E., Dierickx C., Karmisholt K.E., Haedersdal M. (2018). Topical brimonidine reduces IPL-induced erythema without affecting efficacy: A randomized controlled trial in patients with facial telangiectasias. Lasers Surg. Med..

[9] Ross E.V., Smirnov M., Pankratov M., Altshuler G. (2006). Intense Pulsed Light and Laser Treatment of Facial Telangiectasias and Dyspigmentation: Some Theoretical and Practical Comparisons. Dermatol. Surg..

[10] McGregor S., Miceli A., Krishnamurthy K. (2019). Treatment of Facial Telangiectases with Glycerin Sclerotherapy. Dermatol. Surg..

[11] Nguyen C., Kuceki G., Birdsall M., Sahni D.R., Sahni V.N., Hull C.M. (2024). Rosacea: Practical Guidance and Challenges for Clinical Management. Rev. Clin. Cosmet. Investig. Dermatol..

[12] Yap F.H.X., Kumarasinghe S.P. (2017). Brimonidine for treatment of telangiectasia of dermatomyositis. Australas. J. Dermatol..

[13] Mello Netto B.A.S.D., Beiriz Y.D.R., Bonatto A.C., Maciel G.S.B., de Almeida L.R., Corassa J.M. (2020). Uso de tartarato de brimonidina para resolução de matting telangiectásico: Relato de caso. J. Vasc. Bras..

[14] Maliyar K., Abdulla S.J. (2022). Dermatology: How to manage rosacea in skin of colour. Drugs Context.

[15] van Zuuren E.J., Fedorowicz Z., Tan J., van der Linden M.M., Arents B.W., Carter B., Charland L. (2019). Interventions for rosacea based on the phenotype approach: An updated systematic review including GRADE assessments. Br. J. Dermatol..

[16] Yepuri V., Patil A.D., Fritz K., Salavastru C., Kroumpouzos G., Nisticò S.P., Piccolo D., Sadek A., Badawi A., Kassir M. (2021). Light-Based Devices for the Treatment of Facial Erythema and Telangiectasia. Dermatol. Ther..

[17] Goldman M.P., Eckhouse S. (1996). Photothermal sclerosis of leg veins. ESC medical systems, LTD photoderm VL Cooperative Study Group. Dermatol. Surg..

[18] Kim B.Y., Moon H.-R., Ryu H.J. (2018). Comparative efficacy of short-pulsed intense pulsed light and pulsed dye laser to treat rosacea. J. Cosmet. Laser Ther..

[19] Kapicioglu Y., Sarac G., Cenk H. (2019). Treatment of erythema to telangiectatic rosacea, facial erythema, and facial telangiectasia with a 577-nm pro-yellow laser: A case series. Lasers Med. Sci..

[20] Stier M.F., Glick S.A., Hirsch R.J. (2008). Laser treatment of pediatric vascular lesions: Port wine stains and hemangiomas. J. Am. Acad. Dermatol..

[21] Babilas P., Schreml S., Szeimies R.-M., Landthaler M. (2010). Intense Pulsed Light (IPL): A Review. Lasers Surg. Med..

[22] Nistico S.P., Silvestri M., Zingoni T., Tamburi F., Bennardo L., Cannarozzo G. (2021). Combination of Fractional CO2 Laser and Rhodamine-Intense Pulsed Light in Facial Rejuvenation: A Randomized Controlled Trial. Photobiomodul. Photomed. Laser Surg..

[23] Piccolo D., Di Marcantonio D., Crisman G., Cannarozzo G., Sannino M., Chiricozzi A., Chimenti S. (2014). Unconventional use of intense pulsed light. Biomed. Res. Int..

[24] Anzengruber F., Czernielewski J., Conrad C., Feldmeyer L., Yawalkar N., Häusermann P., Cozzio A., Mainetti C., Goldblum D., Läuchli S. (2017). Swiss S1 guideline for the treatment of rosacea. J. Eur. Acad. Dermatol. Venereol..

[25] McCoppin H.H.H., Goldberg D.J. (2010). Laser Treatment of Facial Telangiectases: An Update. Dermatol. Surg..

[26] Ammirati C.T., Carniol P.J., Hruza G.J. (2001). Laser Treatment of Facial Vascular Lesions. Facial Plast. Surg..

[27] Vladimir F., Jorgaqi E., Byzhyti M. (2021). Our experience in the treatment of hemangioma with intense pulsed light laser: A 10 year study in Albania. Dermatol. Ther..

[28] Peterson J.D., Friedmann D.P., Abdo K., Cahana Z. (2020). Efficacy and Safety of Intense Pulsed Light With a KTP/PDLlike Filter for the Treatment of Facial Telangiectasias. J. Drugs Dermatol. JDD.

[29] Piccolo D., Crisman G., Kostaki D., Cannarozzo G., Sannino M., Chimenti S. (2016). Rhodamine intense pulsed light versus conventional intense pulsed light for facial telangiectasias. J. Cosmet. Laser Ther..

[30] Goldman M.P., Weiss R.A., Weiss M.A. (2005). Intense pulsed light as a nonablative approach to photoaging. Dermatol. Surg..

[31] Schroeter C.A., Haaf-von Below S., Neumann H.A.M. (2005). Effective treatment of rosacea using intense pulsed light systems. Dermatol. Surg..

[32] Bennardo L., Patruno C., Zappia E., Tamburi F., Sannino M., Negosanti F., Nisticò S.P., Cannarozzo G. (2022). Combination of Specific Vascular Lasers and Vascular Intense Pulsed Light Improves Facial Telangiectasias and Redness. Medicina.

[33] Husain Z., Alster T.S. (2016). The role of lasers and intense pulsed light technology in dermatology. Clin. Cosmet. Investig. Dermatol..

[34] Murphy M.J., Torstensson P.A. (2014). Thermal relaxation times: An outdated concept in photothermal treatments. Lasers Med. Sci..

[35] Raulin C., Greve B., Grema H. (2003). IPL technology: A review. Lasers Surg. Med..

[36] Tsunoda K., Akasaka K., Akasaka T., Amano H. (2018). Successful treatment of erythematotelangiectatic rosacea with intense pulsed light: Report of 13 cases. J. Dermatol..

[37] Gade A., Vasile G.F., Rubenstein R. (2024). Intense Pulsed Light (IPL) Therapy.

[38] Bjerring P., Christiansen K., Troilius A. (2001). Intense pulsed light source for treatment of facial telangiectasias. J. Cutan. Laser Ther..

[39] Williams N.M., Rajabi-Estarabadi A., Aigen A.R. (2020). Use of Intense Pulsed Light Versus Pulsed-Dye Laser in the Treatment of Truncal Telangiectasia. Dermatol. Surg..

[40] Tanghetti E.A. (2011). Split-face randomized treatment of facial telangiectasia comparing pulsed dye laser and an intense pulsed light handpiece. Lasers Surg. Med..

[41] Campolmi P., Bonan P., Cannarozzo G., Bruscino N., Troiano M., Prignano F., Lotti T. (2011). Intense pulsed light in the treatment of non-aesthetic facial and neck vascular lesions: Report of 85 cases. J. Eur. Acad. Dermatol. Venereol..

[42] Gan H., Yue B., Wang Y., Lu Z. (2018). Treatment of facial telangiectasia with narrow-band intense pulsed light in Chinese patients. J. Cosmet. Laser Ther..

[43] Luo Y., Luan X., Zhang J., Wu L., Zhou N. (2020). Improved telangiectasia and reduced recurrence rate of rosacea after treatment with 540 nm-wavelength intense pulsed light: A prospective randomized controlled trial with a 2-year follow-up. Exp. Ther. Med..

[44] Arsiwala S.Z., Arsiwala N. (2023). Role of Dermoscopy in Laser Therapy. Indian Dermatol. Online J..

[45] Onizuka K., Tsuneda K., Shibata Y., Ito M., Sekine I. (1995). Efficacy of flashlamp-pumped pulsed dye laser therapy for port wine stains: Clinical assessment and histopathological characteristics. Br. J. Plast. Surg..

[46] Fiskerstrand E.J., Svaasand L.O., Kopstad G., Dalaker M., Norvang L.T., Volden G. (1996). Laser treatment of port wine stains: Therapeutic outcome in relation to morphological parameters. Br. J. Dermatol..

[47] Angermeier M.C. (1999). Treatment of facial vascular lesions with intense pulsed light. J. Cutan. Laser Ther..

[48] Retamar R.A., Chames C., Pellerano G. (2014). Treatment of linear and spider telangiectasia with an intense pulsed light source. J. Cosmet. Dermatol..

[49] Li N., Han J., Hu D., Cheng J., Wang H., Wang Y., Yang X., Liu J., Li T., Zhao W. (2018). Intense pulsed light is effective in treating postburn hyperpigmentation and telangiectasia in Chinese patients. J. Cosmet. Laser Ther..

